# Intraperitoneal Mesenchymal Cells Promote the Development of Peritoneal Metastasis Partly by Supporting Long Migration of Disseminated Tumor Cells

**DOI:** 10.1371/journal.pone.0154542

**Published:** 2016-05-03

**Authors:** Joji Kitayama, Hironori Yamaguchi, Hironori Ishigami, Keisuke Matsuzaki, Naohiro Sata

**Affiliations:** 1 Department of Gastrointestinal Surgery, Jichi Medical University, Tochigi, Japan; 2 Department of Surgical Oncology, University of Tokyo, Tokyo, Japan; 3 Kanamecho Hospital, Tokyo, Japan; University of Kentucky College of Medicine, UNITED STATES

## Abstract

The human peritoneal cavity contains a small number of free cells of mesenchymal cell lineage. Intraperitoneal mesenchymal cells (PMC) play supportive roles in metastasis formation on the peritoneum. In this study, we found that PMC, when co-cultuerd with human gastric cancer cells, MKN45, enhanced the proliferation of MKN45 when cultured at low, but not high, cellular density. Also, PMC suppressed apoptotic cell death of MKN45 only under low density culture conditions. Time-lapse videoanalysis clearly demonstrated that PMC randomly migrated more vigorously than did MKN45, and strongly enhanced the migration behavior of co-cultured MKN45. In fact, the majority of MKN45 migrated together in direct physical contact with PMC, and the sum of migration lengths from original position of co-cultured MKN45 for 48 hours was approximately 10 times longer than that of MKN45 cultured alone. Our data suggest that enhanced migration can increase the chance of direct contact or positional proximity among sparcely distributed MKN45, which may bring survival advantages to tumor cells. This may be one of the important mechanisms of peritoneal metastasis, since only a small number of tumor cells are considered to be disseminated in the early step of metastasis formation on the peritoneum.

## Introduction

The peritoneal cavity is a common target of metastatic gastrointestinal and ovarian cancer cells [1–3]. Although, peritoneal metastasis is likely to develop from intraperitoneal free tumor cells exfoliated from the serosal surface of primary tumors [4,5], the mechanisms leading to peritoneal metastasis have not been fully elucidated. The peritoneal cavity is the largest free space in the human body. It contains a large amount of adipose tissue and is covered by mesothelium, which has a smooth and nonadhesive surface that facilitates intracoelomic movement. In addition, the peritoneal cavity physiologically contains various free cells which may have positive roles in metastasis formation, since increasing evidence suggests that tumor metastasis is critically influenced by non-malignant cells in the tumor microenvironment [6–8].

Many studies have shown that cells of mesothelial lineage can be successfully obtained by in vitro culture of peritoneal or pleural effusion [9–11]. Recently, we have suggested that these cells originate from a small number of free cells with CD90(+)CD45(-) phenotype which vigorously grow in vitro with morphological similarity to mesothelial cells. Then, we named the cells as mesothelial-like cell (MLC) and evaluated their biological functions. Interestingly, co-transfer of the MLC enhanced metastasis formation of human gastric cancer cells on the peritoneum in nude mice via the active production of fibrous stroma [3] as well as potent inhibition of T cell-mediated immunity [12]. In 2002, Foley-Comers et al initially reported the similar cells in peritoneal cavity which had a physiological function of regeneration of mesothelium [13] and later they described the cells as mesothelial progenitor cells [14]. The MLC in our previous study appear to be same cells as theirs. Here, in this study, we renamed the cells as intraperitoneal mesnchymal cells (PMC) and further investigated their roles in the process of development of peritoneal metastasis, especially from the aspect of the effects on motility of disseminated tumor cells using time-lapse videoanalysis.

## Materials and Methods

### Cells and Reagents

The human gastric cancer cell line, MKN45, was obtained from Riken (Tsukuba Japan, Catalogue No. RCB1001), and maintained in Dulbecco’s Modified Eagle Medium (DMEM) supplemented with 10% (w/v) fetal bovine serum (FBS) (Sigma, St. Louis, MO), 100 units/ml penicillin and 100 mg/ml streptomycin (Life Technologies, Inc., Grand Island, NY). Intraperitoneal mesenchymal cells (PMC) were obtained from peritoneal lavage fluid recovered from patients who underwent abdominal surgery in the Departments of Surgical Oncology or Kanamecho Hospital as described previously [12]. Written informed consent was obtained from all patients. After centrifugation of ascites or peritoneal lavage fluid at 1500 rpm for 15 min, the pellets were resuspended in PBS + 0.02% (w/v) EDTA and overlaid on Ficoll-Hypaque solution (Pharmacia Biotech, Piscataway, NJ). After centrifugation at 3000 rpm for 10 min, the intermediate layer was taken and washed twice with PBS + 0.02% (w/v) EDTA. These cells were cultured in DMEM supplemented with 10% (w/v) FCS, 100 units/ml penicillin and 100 μg/ml streptomycin in Type I collagen-coated plates or flasks (Iwaki, Tokyo Japan). After reaching confluence, the cells were removed by treatment with 0.02% (w/v) EDTA and trypsin, and passaged. After 2~3 weeks of culture, more than 95% of cells showed a CD45(-)CD90(+) phenotype, which were used for the experiments.

FITC-conjugated annexin V was purchased from Biolegend (SanDiego, CA), PE-conjugated mAbs to CD326 (EpCAM) and 7-amino-actinomycin D (7-AAD) were from Miltenyi Biotec (Auburn, CA).

This study was carried out in accordance with the Declaration of Helsinki and was approved by the Institutional Review Board of the University of Tokyo.

### In vitro evaluation of MKN45 proliferation and apoptosis

PMC (1x104) at 2~3 passages were suspended in 1 ml DMEM containing 10% FBS and seeded in Type I collagen-coated 6-well plastic plates (Iwaki, Tokyo, Japan). After an hour, various numbers of MKN45 (1x103~1x105) suspended in 1 ml of the same medium were added and cultured for several days at 37°C in 5% CO_2_. As control, the same numbers of MKN45 were cultured alone without PMC. In low cellular density condition (1×10^3^~5×10^3^ MKN45 cells/well), the culture was continued up to 14 days with half medium change every 2~3 days. In some experiments, the same number of PMC were seeded in culture inserts and transferred onto the 6-well plates containing the same number of MKN45. In some experiments, PMC were separately cultured in inserts with 0.4-μm pores to inhibit direct contact with MKN45. At the end of culture, the entire cells were harvested using 0.02% (w/v) EDTA and trypsin, washed, and stained with FITC-conjugated annexin V, PE-conjugated mAbs to CD326, and 7-AAD. Then, the cells were resuspended in 0.5ml PBS + 0.02% (w/v) EDTA, and the density of MKN45 in each sample was evaluated using flowcytometry. In short, the entire cells in each suspension were acquired for 30 seconds, and the number of cells with the annexin V(-) CD326(+) 7AAD(-) phenotype in each acquisition were gated and counted as alive MKN45 cells, which enabled the exclusion of dead cells and contaminating CD326(-) PMC. Then, the percentage of alive MKN45 co-cultured with PMC was calculated against that of MKN45 cultured alone for the same culture periods. To determine the apoptotic index of MKN45, the percentage of annexin V(+) 7AAD(+) cells was calculated in gated CD326(+) cells.

### Motility analysis

As described previously, 1×10^4^ MKN45 were seeded with or without the same number of PMC in type I collagen-coated dishes with 3.5 cm diameter, and cell movements were monitored for 48 hours using a Biostudio system (Nikon Engineering, Kanagawa, Japan). In short, photo images of a selected field were taken every 5 min for 48 hours, and movies were constructed in a time lapsed manner using Cell Image Viewer software (Nikon Engineering, Kanagawa, Japan). On each video movie, 10~17 MKN45 cells were randomly selected in a field and the locations of each cell were chronologically plotted using image analysis software, DIPP-Motion V2D (DITECT, Tokyo, Japan). Then, the motions of each cell were monitored and the total length of migration from original position in each 5 min. was summed for 48 hours in each MKN45 using the software.

### Statistical analysis

The results were analyzed by paired Student’s t test or Wilcoxon’s test where appropriate. Results are given as means and differences with P <0.05 were considered significant.

## Results

### Characterization of PMC

Cells recovered from the peritoneal cavity were maintained as bulk cultures in 10% FCS + DMEM media on type I collagen coated plates. In all cases, the majority of the cells were CD45(+)CD90(-) leukocytes before the culture. However, under the culture condition, the CD45(-)CD90(+) cells grew vigorously in an adherent manner and became the major component. After 2~3 weeks of culture, more than 95~99% of cells showed a CD45(-)CD90(+) phenotype, which were used for the experiments (S1 Fig). In addition to CD90, those cells were positive for CD73, CD105, CD166 which were all reported to be a marker of mesenchymal stem cell. Those cells were also positive for mesenchymal markers, vimentin and type 1 collagen. Interestingly, they were positive for cytokeratin and calreticulin which may suggest the mesothelial differentiation. In contrast, those cells were totally negative for hematopoietic markers including CD14 and CD45 (S2 Fig) As described previously [3,12], we defined the cells as “mesothelial-like cell” from their morphology. In this study, however, we renamed the cells as intraperitoneal mesenchymal cells (PMC).

### PMC increased proliferation and reduced apoptosis of MKN45 co-cultured at low cellular density

**Fig 1:**
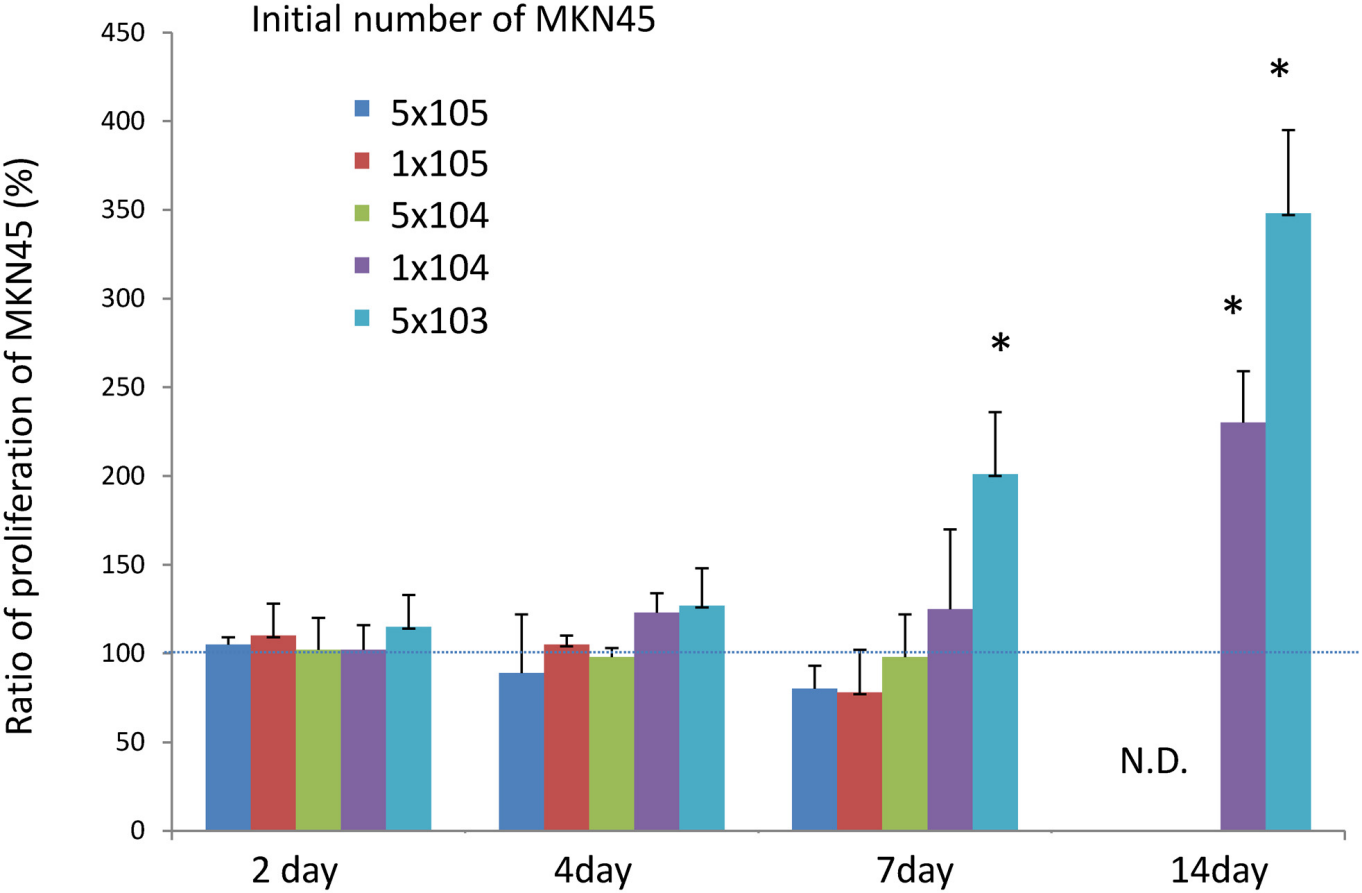
Effect of PMC on in vitro growth of MKN45. MKN45 (5x10^3^-1x10^5^) were cultured in 6-well culture plates with or without 1x10^4^ PMC. Two to 14 days later, whole cells were harvested using 0.02% EDTA + trypsin, immunostained with FITC-conjugated annexin V, PE-conjugated mAbs CD326, and 7AAD, and resuspended in the same volume (0.5ml) of PBS+0.02% EDTA. Then, the cells were acquired for 30 seconds with FACS, and the number of cells with annexinV(-), CD326(+), 7AAD(-) were counted as alive MKN45 cells in each sample. The percentages of the numbers of MKN45 co-cultured with PMC were calculated against those of MKN45 cultured alone. Data show mean±SD of 3 different experiments. N.E: not evaluated.

First, we calculated the number of MKN45 cells cultured with or without PMC using FACS to examine whether PMC can affect the in vitro proliferation of MKN45. When the cultures were started from 5x10^4^ or 1x10^5^ MKN45, the growth of MKN45 did not show a significant difference by the addition of PMC, although the number of MKN45 tended to be lower at 7 days, possibly due to overgrowth (Fig 1). However, when the initial number of MKN45 was reduced to 1x10^4^ or 5x10^3^ cells /well, the growth of MKN45 was increased by PMC at day 7 or later. The number of MKN45 cultured with PMC for 14 days reached 230±29% and 378±47% of that of MKN45 cultured alone, respectively. This suggests that PMC enhance the growth of MKN45 when cultured at low, but not high, cellular density.

**Fig 2:**
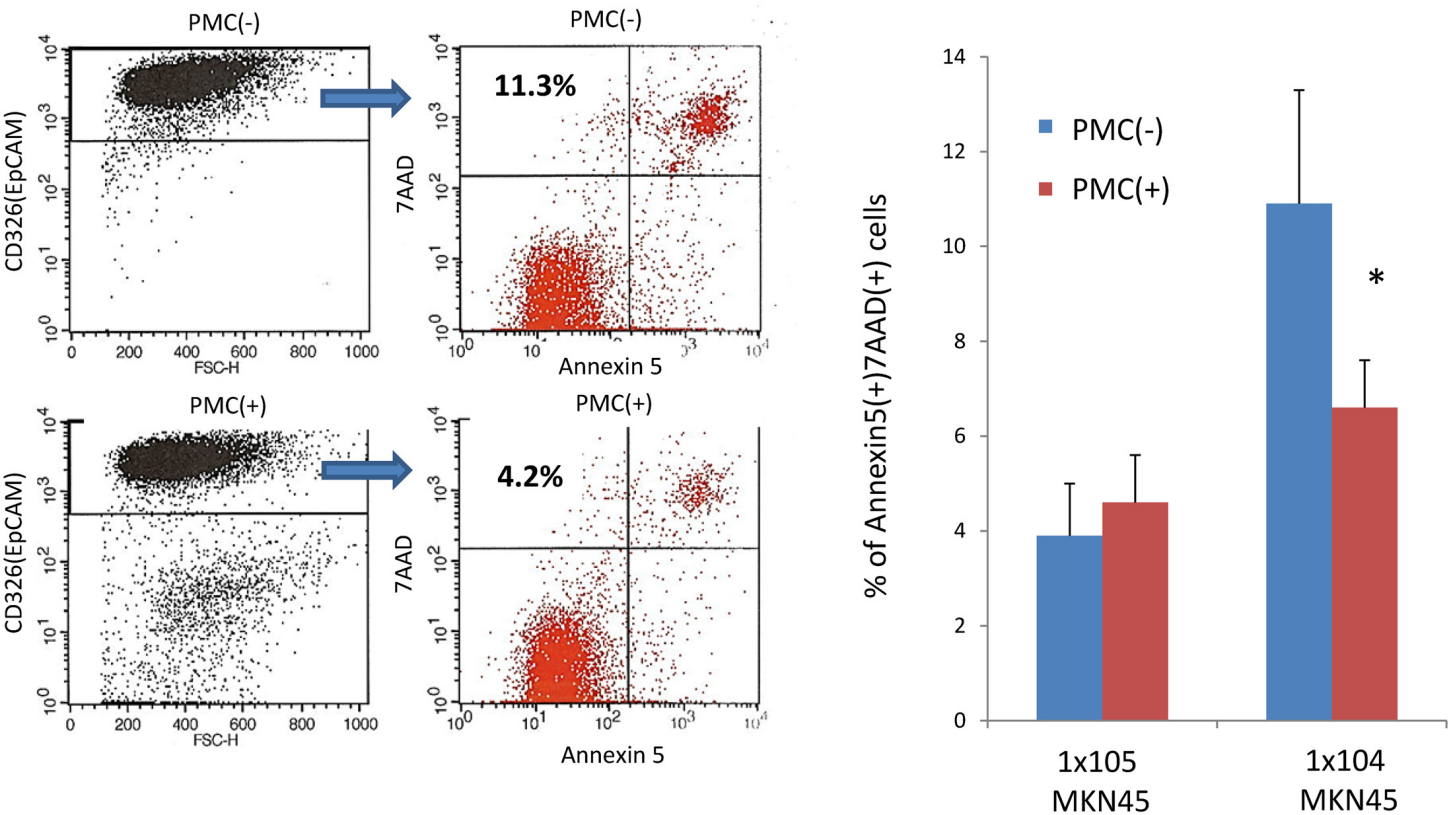
Effect of PMC on apoptosis of MKN45. (A) MKN45 (1x104 or 1x105) was cultured with or without PMC (1x104) in 6-well dish for 48 hours. The cells were immunostained as described in the legend of Fig 1, and the percentages of annexinV(+) 7AAD(+) cells in CD326(+) population were calculated. Data show mean±SD of 3 different experiments. (B) Representative FACS profiles of MKN45 (1x104) cultured with (lower panels) or without (upper panels) 1x104 PMC.

Next, we examined the ratio of MKN45 that showed apoptotic cell death at 48 hours. As shown in Fig 2A, when 1x10^5^ MKN45 were co-cultured with PMC, the ratio of apoptotic MKN45 was not affected by PMC (3.9±1.2% vs 4.6±0.9%, n = 3) In contrast, when one tenth of the number of MKN45 (1x10^4^) were co-cultured with PMC, the percentage of apoptotic MKN45 was increased (10.9±2.4%, n = 3), which was significantly reduced by the addition of PMC (6.6±2.2%, n = 3, p<0.05). Fig 2B shows a representative FACS profile and the ratio of annexin V(+) 7AAD(+) phenotype in CD326(+) MKN45 which were cultured at low cellular density.

### Effect of PMC on MKN45 behavior is not mediated by soluble factor

**Fig 3:**
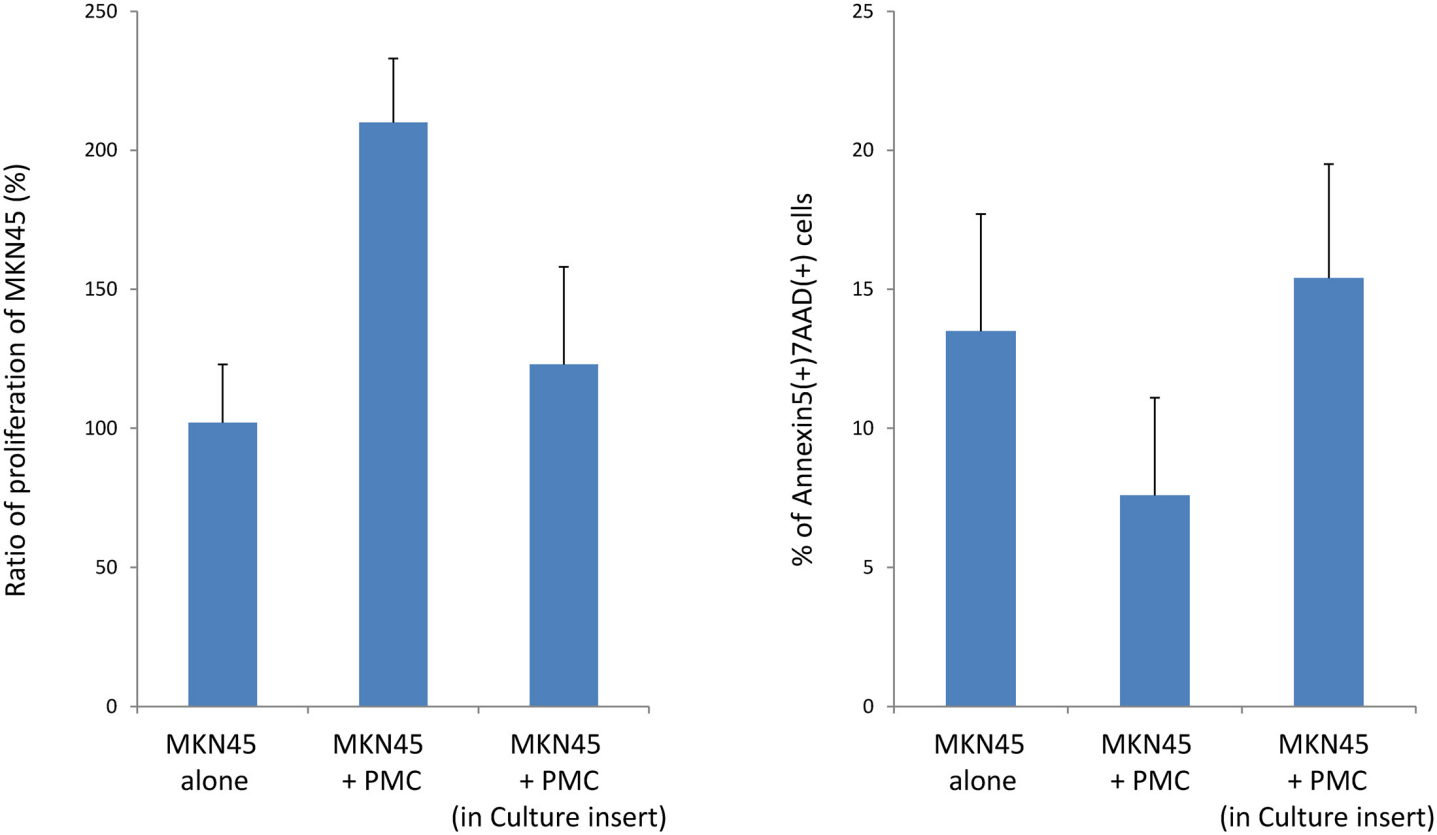
Effect of PMC on MKN45 is not mediated by soluble factors. MKN45 (1x104) were cultured with or without PMC (1x104) in 6-well culture dishes. In some wells, PMC were separately cultured in inserts with 0.4-μm pores to inhibit direct cell-cell contact. Then the ratio of proliferation at 7 days and apoptosis at 2 days of MKN45 were examined as described in the legends of Figs 1 and 2. Data show mean±SEM of 2 different experiments.

We next examined the growth and apoptosis of MKN45 co-cultured with PMC, avoiding cell-cell contact using a double chamber culture system. As shown in Fig 3A, the number of MKN45 at 7 days was clearly increased by PMC co-cultured in the same well (210±23%), while it was not significantly increased by PMC cultured on inserts (123±35%). Consistently, apoptosis of MKN45 was not significantly affected by PMC cultured separately (13.5±4.2% vs 15.4±4.1%) (Fig 3B). Moreover, the addition of the culture medium of PMC did not affect the ratio of apoptosis of MKN45 (S3 Fig). This indicates that the effects of PMC on the growth of MKN45 were not mediated by soluble factors, but were mostly dependent on direct contact between the two cell types.

### PMC vigorously move and support long migration of MKN45 via direct physical contact

**Fig 4:**
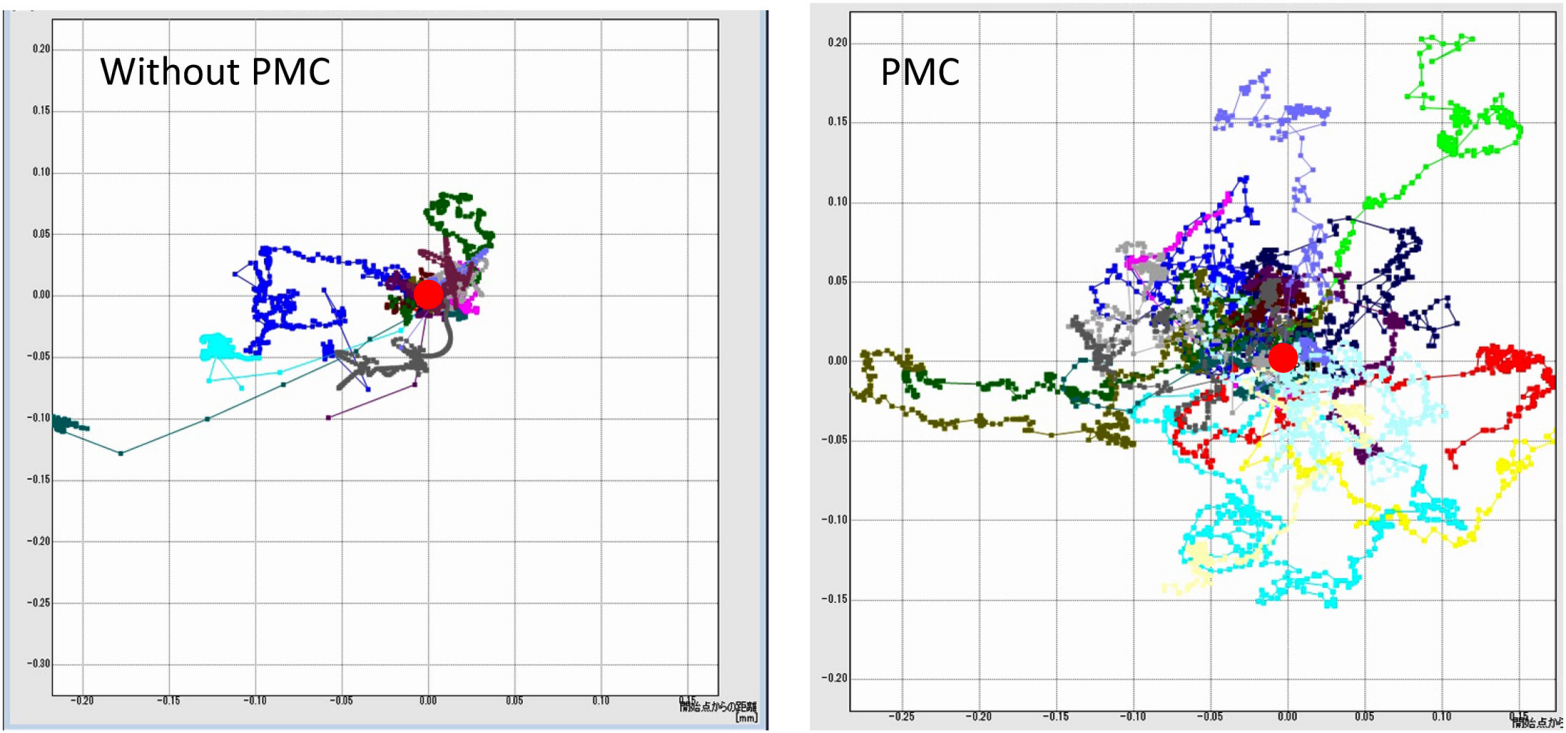
Tracks of MKN45 cultured with or without PMC. MKN45 (1x104) were seeded with (B) or without (A) 1x104 PMC in 3.5 cm diameter dishes and cultured on the stage of a Biostudio system in 5% CO2 incubator for 48 hours. During culture, photo images of a selected field were taken every 5 min. and the locations were separately marked for randomly selected MKN45 using image analysis software, DIPP-Motion V2D. Then, the tracks of migration of these MKN45 were expressed with the initial position fixed (red circles). Data show the tracks of 16 MKN45 cells in one of the 3 different experiments.

**Fig 5:**
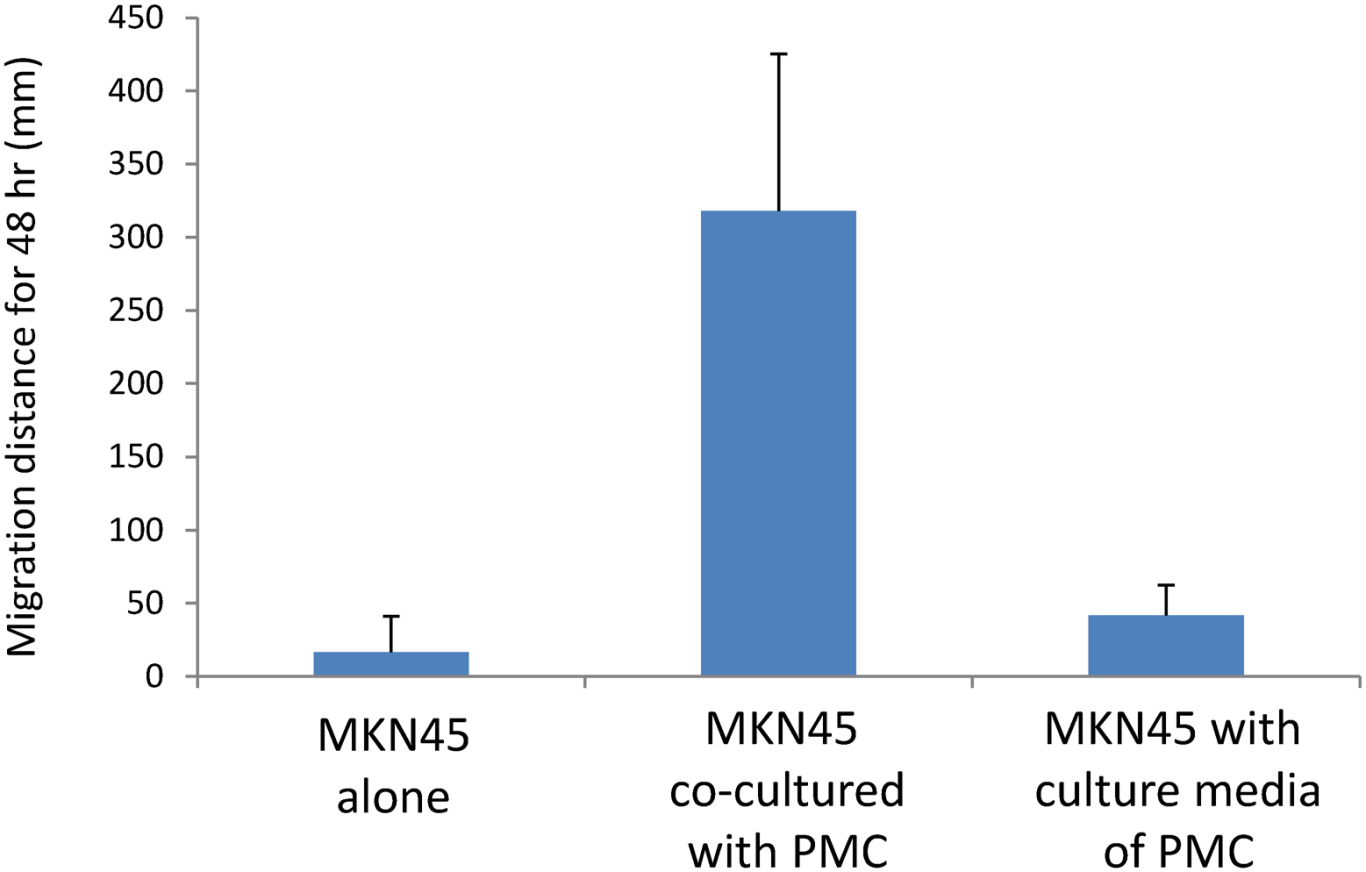
Length of migration of MKN45 cultured with or without PMC. MKN45 were cultured as described in the legend of Fig 4. In these video movies, the distances of displacement in each time point were summed in randomly selected MKN45 using DIPP-Motion V2D software. Data show mean±SD of 43 cells in 3 different experiments.

Then, we evaluated the two-dimensional movements of MKN45 cells on plastic plates. MKN45 were cultured with or without PMC under the same low cell density in dishes with 3.5 cm diameter, and the migration patterns of MKN45 were monitored for 48 hours using time lapse video analysis. Representative movies are shown in S1 and S2 Videos. In these movies, MKN45 and PMC were easily distinguished as small bright cells and large adherent cells, respectively. As shown in S1 Video, most of the MKN45 moved around in small areas close to their initial position (within circles of less than 100 μM diameter), except that were detached and flew away, possibly due to the convection current of the culture medium. In comparison, when MKN45 were cultured with PMC, PMC exhibited markedly more vigorous migration than did MKN45 (S2 Video). More interestingly, MKN45 attached to the co-cultured PMC moved together with the physical association with PMC and migrated obviously longer distance as compared with MKN45 cultured alone. In other words, tumor cells “piggy back” on the PMCs to migrate. Fig 4 shows a comparison of the 16 migration tracks of randomly selected MKN45 in a representative experiment. In 3 different experiments, the total length of migration of MKN45 co-cultured with PMC for 48 hours was almost 10 times longer than that of MKN45 cultured alone (357.7±189.6 mm vs 38.3±42.7 mm, n = 43, p<0.001) (Fig 5). Finally, we added the medium that cultured PMC for 48 hours to MKN45 and examined the motility of MKN45. However, the migration distance of these MKN45 for 48 hours was 55.9±55.1 mm (n = 17), which was not significantly different from that of MKN45 without culture medium (Fig 5). This indicates that direct contact of MKN45 with PMC is essential to enhance the migration behavior of MKN45.

## Discussion

Solid tumors are composed not only of tumor cells but also of non-malignant cells, and the complex interaction among these cells is thought to enable progression and metastasis [6,15]. In previous studies, we demonstrated that PMC assist metastasis formation in the peritoneal cavity by forming a tumor permissive microenvironment [3,12]. Here, in this study, we found an additional mechanism of PMC to promote peritoneal metastasis. In the first step, we asked whether PMC can enhance the in vitro growth of MKN45, and found that the presence of PMC significantly increased the proliferation and reduced apoptosis by PMC only when MKN45 were cultured at low density. This suggests the possibility that intercellular communication that critically affects the destiny of MKN45 occurs in culture conditions of low, but not high, cellular density.

Intercellular communication is closely related to cell motility. Thus, we then used time lapse video analysis and investigated the movement of MKN45 co-cultured with PMC at the same low culture density during the early culture period. These video movies clearly demonstrated that PMC were highly motile on plastic plates as compared with MKN45, and that PMC enabled long migration of co-cultured MKN45. In fact, most of the MKN45 attached to PMC were actually “carried” by the moving PMC, whereas MKN45 that did not meet PMC showed the same pattern of migration as those cultured alone. These “lonely” MKN45 might finally undergo apoptotic cell death. From these findings, it is speculated that the enhanced migration of MKN45 causes an increased chance of physical interaction of sparsely distributed MKN45 cells, which can produce juxtacrine signaling to inhibit apoptosis and bring an advantage of cell growth, as described elsewhere [16]. Indeed, movies of the co-culture condition suggested that some of the MKN45 that moved a long distance met together with other MKN45 and formed a small cluster within 48 hours. In contrast, when MKN45 were cultured at high density, they easily made the intercellular contact even without PMC (data not shown), which may be the reason why the growth of MKN45 was not significantly affected by the presence of PMC.

Previous studies have indicated that cancer associated fibroblasts (CAF) stimulates cancer cell motility or invasion by producing soluble factors such as Hepatocyte growth factor (HGF) [17], Transforming growth factor (TGF)-β [18,19], CXCL12 [20]. In our experiments, however, the enhanced migrations as well as growth of MKN45 were considered to be totally mediated by direct contact between PMC and MKN45, because addition of the culture medium of PMC did not affect the behavior of MKN45. This is consistent with the results of recent studies which have suggested that direct interaction with fibroblasts is important to enhance tumor cell invasion [21,22] Although dependent on the experimental conditions or characteristics of tumor cells, our data suggest that physical contact with mesenchymal cells critically regulates the migration and invasive behavior of tumor cells.

Peritoneal recurrence often develops even after curative resection of abdominal malignancies [23–25]. In those cases, metatasatic foci are considered to develop from undetectable tumor cells disseminated in the peritoneal cavity. Our in vitro study provides the clear evidence that PMC support the colony formation and proliferation of sparsely distributed tumor cells. This observation seems to be clinically important in the mechanism of metastasis formation in peritoneum, because only a small number of tumor cells are supposed to be disseminated in the early step of peritoneal metastasis. Pharmacological inhibition of PMC during postoperative adjuvant chemotherapy may be another effective strategy to inhibit peritoneal recurrence of abdominal malignancy.

## Supporting Information

S1 Fig Intraperitoneal free cells were recovered from peritoneal lavages or ascites recovered at laparotomy from 3 patients with gastrointestinal cancer and stained with FITC-conjugated anti-CD45 mAb, PE-conjugated anti-CD90 mAb and 7-AAD. After the bulk culture for 16 to 21 days, the cells were recovered and immunostained with same method. Number show the percentages of CD45(-)CD90(+) cells in 7-AAD(-) cell population.
(TIF)

S2 Fig Antigen expression pattern of the typical PMC. Cells recovered from the peritoneal cavity of a patient with gastric cancer were cultured for 18 days and their phenotypes were examined by FACS. The cells were detached from culture plate and fixed and permeabilized using BD Cytofix/Cytoperm (Becton-Dickinson, San Jose, CA) before immunostaining with each mAbs. Green lines denote the fluorescent profiles of the indicated antigens and filled lines correspond to negative controls.
(TIF)

S3 Fig MKN45 (1x104) were cultured in 6-well culture dish with normal medium or culture medium of PMC (1x104) for 3days. After 48 hour incubation, MKN45 were harvested and stained with annexinV and percentages of apoptotic cells were calculated. Data show mean±SEM in 2 different experiments.
(TIF)

S1 Video MKN45 (1x104) were cultured without same number of PMC in dishes with 3.5 cm diameter, and the migration patterns of MKN45 were monitored for 48 hours using using a Biostudio system (Nikon Engineering, Kanagawa, Japan). Photo images of a selected field were taken every 5 min for 48 hours, and movies were constructed in a time lapsed manner using Cell Image Viewer software (Nikon Engineering, Kanagawa, Japan). On each video movie, 16 MKN45 cells were randomly selected in a field and the locations of each cell were chronologically plotted using image analysis software, DIPP-Motion V2D (DITECT, Tokyo, Japan).
(WMV)

S2 Video MKN45 (1x104) were cultured with same number of PMC in dishes with 3.5 cm diameter, and the migration patterns of MKN45 were monitored for 48 hours using using a Biostudio system (Nikon Engineering, Kanagawa, Japan). Photo images of a selected field were taken every 5 min for 48 hours, and movies were constructed in a time lapsed manner using Cell Image Viewer software (Nikon Engineering, Kanagawa, Japan). On each video movie, 16 MKN45 cells were randomly selected in a field and the locations of each cell were chronologically plotted using image analysis software, DIPP-Motion V2D (DITECT, Tokyo, Japan).
(WMV)
