# Supplementary figures and images for: Intraperitoneal Mesenchymal Cells Promote the Development of Peritoneal Metastasis Partly by Supporting Long Migration of Disseminated Tumor Cells
Source: PLoS One. 2016 May 3;11(5):e0154542. doi: 10.1371/journal.pone.0154542 (PMC4854412; doi:10.1371/journal.pone.0154542)

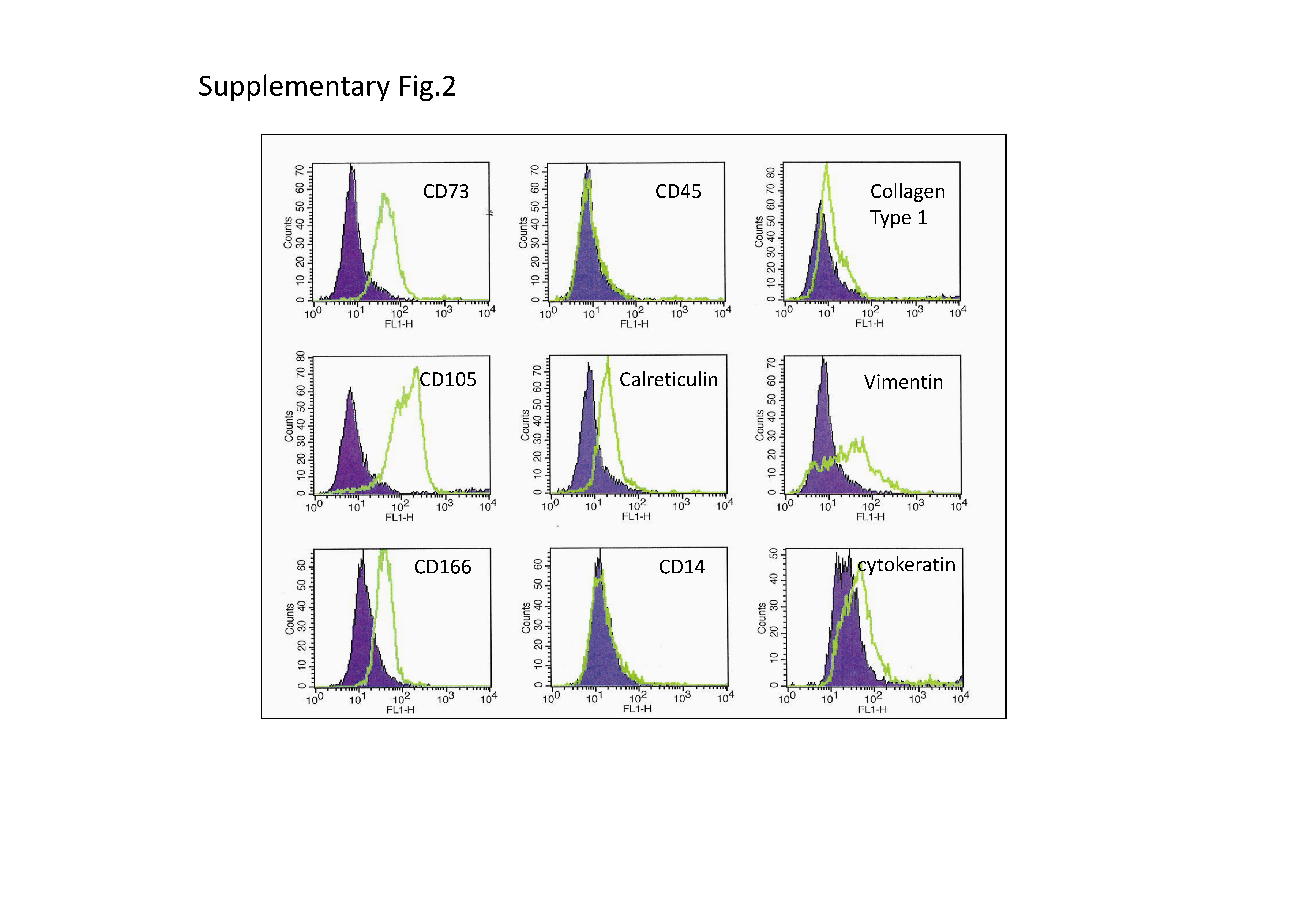

Supplement: S1 Fig — After the bulk culture for 16 to 21 days, the cells were recovered and immunostained with same method. Number show the percentages of CD45(-)CD90(+) cells in 7-AAD(-) cell population. (TIF) [file pone.0154542.s001.tif]

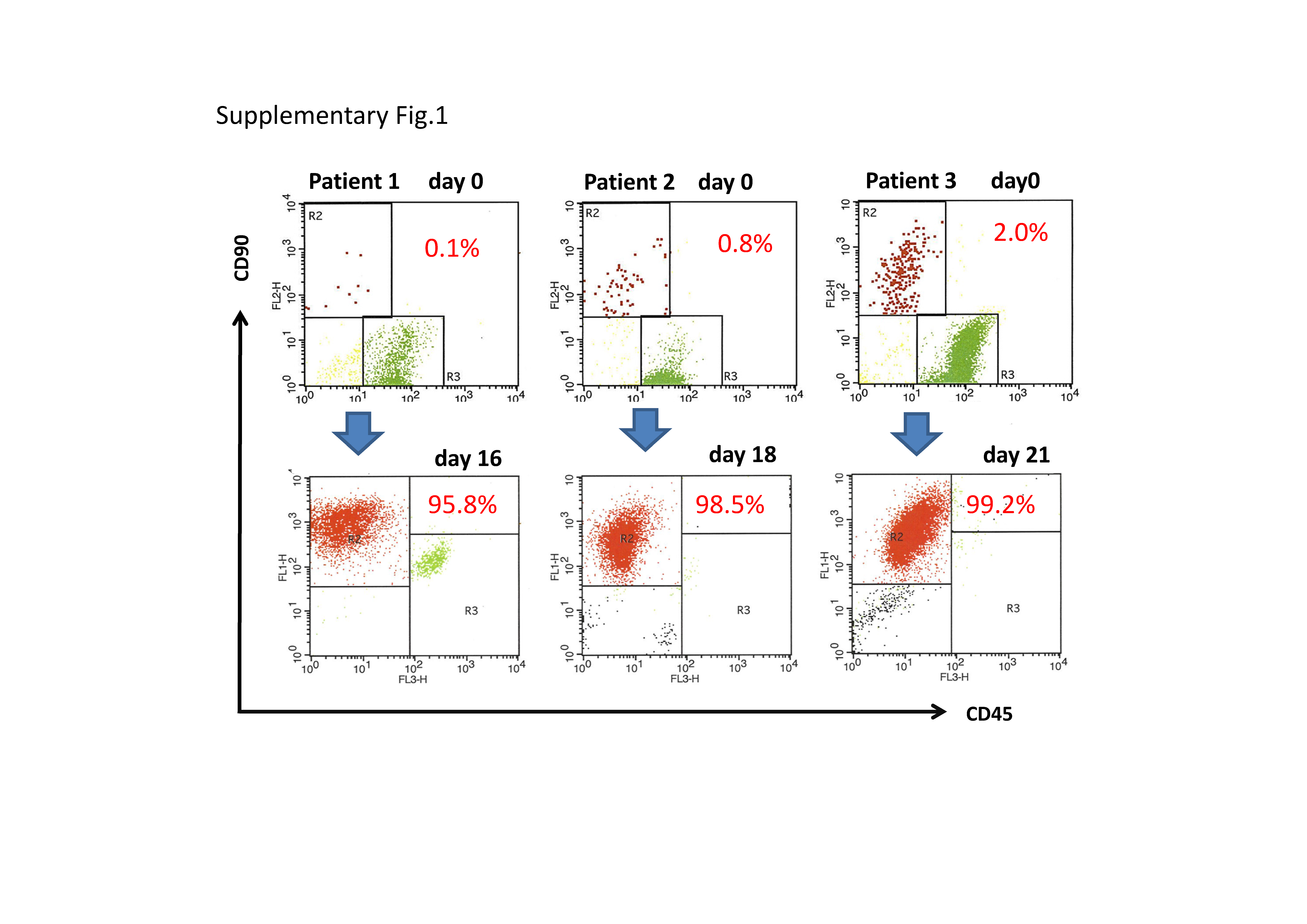

Supplement: S2 Fig — Cells recovered from the peritoneal cavity of a patient with gastric cancer were cultured for 18 days and their phenotypes were examined by FACS. The cells were detached from culture plate and fixed and permeabilized using BD Cytofix/Cytoperm (Becton-Dickinson, San Jose, CA) before immunostaining with each mAbs. Green lines denote the fluorescent profiles of the indicated antigens and filled lines correspond to negative controls. (TIF) [file pone.0154542.s002.tif]

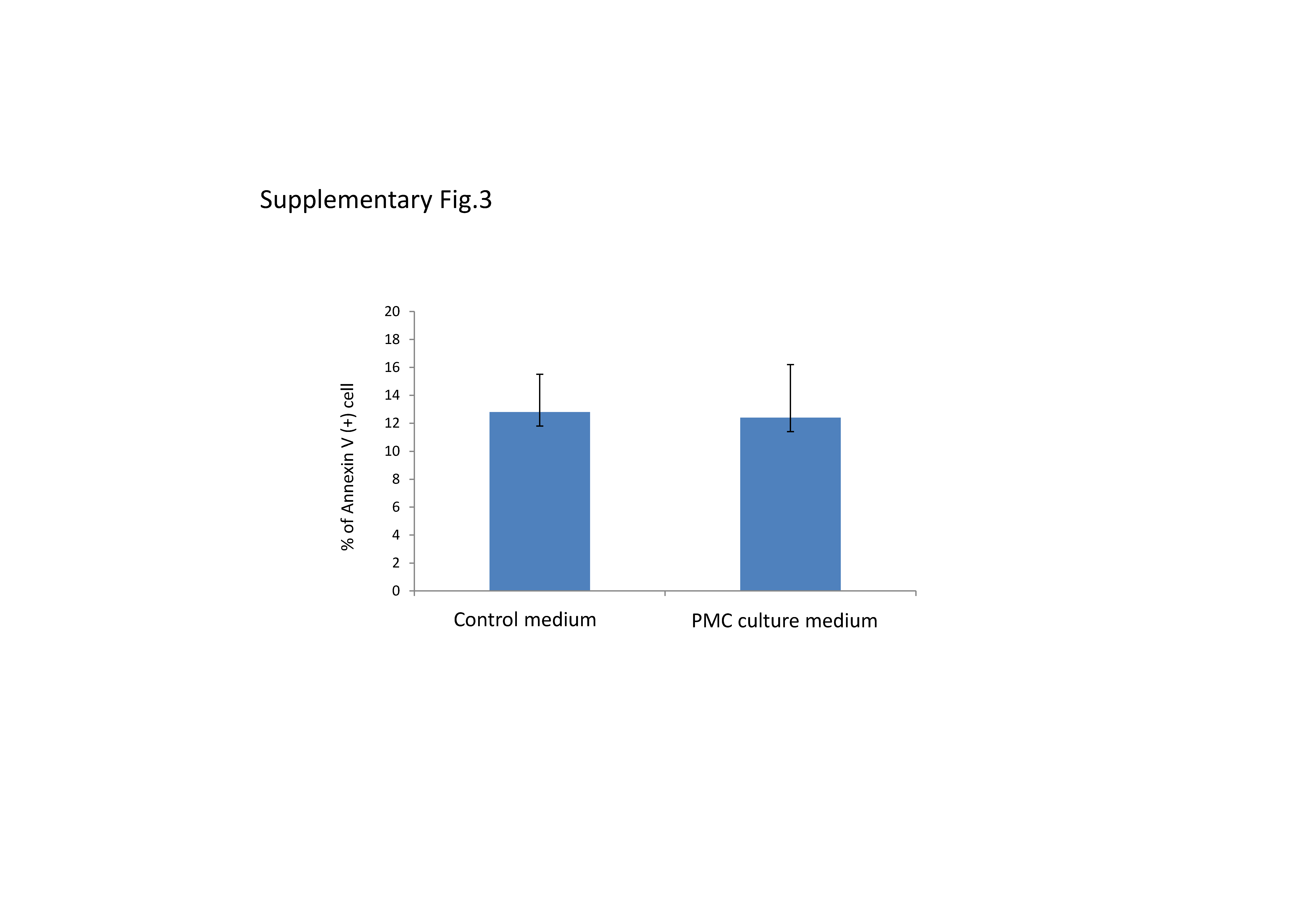

Supplement: S3 Fig — After 48 hour incubation, MKN45 were harvested and stained with annexinV and percentages of apoptotic cells were calculated. Data show mean±SEM in 2 different experiments. (TIF) [file pone.0154542.s003.tif]
